# Editorial: Diet, nutrition, and functional foods for chronic pain

**DOI:** 10.3389/fnut.2024.1456706

**Published:** 2024-09-12

**Authors:** Rowena Field, Fereshteh Pourkazemi, Mohammad Hashem Hashempur, Muthu Thiruvengadam, Kieron Rooney

**Affiliations:** ^1^Faculty of Medicine and Health, The University of Sydney, Sydney, NSW, Australia; ^2^Department of Persian Medicine, School of Medicine, Research Center for Traditional Medicine and History of Medicine, Shiraz University of Medical Sciences, Shiraz, Iran; ^3^Department of Applied Bioscience, College of Life and Environmental Science, Konkuk University, Seoul, Republic of Korea

**Keywords:** diet, nutrition, chronic pain, inflammation, ultra-processed food, functional foods, integrative medicine

Persistent or chronic pain is a global issue affecting approximately 20% of the adult population worldwide (1). Chronic pain is defined as pain persisting beyond the normal tissue healing time, typically lasting more than 3 months, and is regarded as a distinct condition rather than just a symptom of an underlying disease (2). The development of chronic pain remains poorly understood, leading to challenges in identifying definitive treatments for this highly prevalent health issue. Current standards of care emphasize a multifaceted, multidisciplinary approach that addresses the complex interplay between biological, psychological, and social factors (3, 4). However, despite efforts, the implementation of biopsychosocial approaches remains challenging (5), and the effectiveness of available treatments to significantly improve outcomes for patients with chronic pain remains limited. It is crucial that treatment strategies prioritize personalized care and management plans aimed at maintaining patient activity levels and effectively managing pain. As part of comprehensive care plans, a modified diet has recently (6, 7) been recognized as a valuable lifestyle strategy for enhancing pain management outcomes (8).

Current evidence supporting dietary intervention as a part of treatment plans for chronic pain is outlined in two recent systematic reviews with meta-analyses of human studies (7, 12) and one systematic review of preclinical studies (13). Rather than identifying a superior diet, the evidence highlights a broad range of dietary “types” (e.g., Mediterranean) with favorable outcomes for pain. These findings support that common factors among these approaches—such as weight loss, diet quality, and nutrient density—may play a role in modulating pain neurophysiology, providing a general guide applicable to any preferred dietary pattern (7). In contrast to evidence on broader dietary patterns, some research focuses on specific food elements, namely functional foods. For example, evidence suggests that incorporating functional foods such as peppermint, turmeric, ginger, and green tea into dietary modifications can enhance their effectiveness in alleviating pain and promoting overall wellbeing. These benefits may be due to the bioactive compounds in these foods, which have anti-inflammatory and analgesic properties (9–11). Additionally, the evidence underscores the need for further research on the impact of diet on pain experience. Building on the current evidence, this Research Topic presents six papers that provide a variety of evidence demonstrating that diet can influence pain outcomes. Three of these articles were focused on dietary interventional studies for patients with chronic pain (Ciaffi et al.; Sala-Climent et al.; Ward et al.), and two were secondary analyses of genomic data investigating the association of dietary factors with chronic pain (Liu et al.; Dai et al.). One was a cross-sectional study investigating the association of a plant-based diet with migraine headaches (Karimi et al.).

A typical Western diet is characterized by the consumption of ultra-processed foods (UPF). The creation of UPF involves the degradation of the matrix of whole-food ingredients, combined with processed additives designed to restore sensory aesthetics to create hyper-palatable and nutrient-poor foods (14). Deficiency of essential vitamins and minerals may play a role in the development of chronic pain. For example, low Vitamin D levels have been associated with chronic widespread pain presentations (15) and higher levels of the inflammatory biomarker C-reactive protein (CRP) (16). Other nutrients of concern include magnesium, zinc, and vitamin B12 (17, 18). A Western diet high in UPFs is nutrient deficient and high in inflammatory mediators and has been linked to an increased risk of chronic pain (19). Improvements in diet quality and nutrient density are effective pain treatment options (6, 20). This includes both the addition of nutrients needed for optimal nervous system function and the removal of anti-nutrients, such as artificial colors, preservatives, and flavors. A case was also made for the inclusion of phytochemicals (naturally occurring molecules in plants such as curcumin and resveratrol) that have anti-inflammatory and antioxidant actions and may be of therapeutic potential in chronic pain conditions (13, 21). Sala-Climent et al. used a Mediterranean diet to add nutrient density and remove potentially inflammatory mediators, such as gluten and vegetable oils, by removing UPF and focusing on whole foods and extra virgin olive oil, demonstrating improved pain, sleep, and metabolic markers. Similarly, when investigating the effect of a plant-based diet on migraine, Karimi et al. found improvement with a whole-food approach but not with ultra-processed foods.

Most UPFs are refined carbohydrates with a high glycemic index, resulting in large blood glucose excursions and a concomitant large insulin response (22). Their removal from the diet can result in a reduction in total carbohydrate intake, blood glucose excursions, and the subsequent requirement for insulin. A recent randomized clinical trial on chronic pain that compared reducing UPF to the same diet plus reducing carbohydrate (ketogenic diet) found a significant reduction in pain in both groups. The reduction in UPF alone resulted in a reduction in total carbohydrate (209 g/day to 152 g/day) even though this was not an intended consequence (6). Chronically elevated blood glucose levels and the resultant hyperinsulinemia have been linked to pain conditions such as fibromyalgia (23, 24) and glial-mediated sensitization of nociceptors in diabetic neuropathy (25). They also generate advanced glycation end products (AGES) which sensitize nociceptors (26), stiffen tendons (27), and activate enzymes within the cartilage matrix, resulting in damage and inflammation (28). Ciaffi et al. postulated that a very low-calorie ketogenic diet (VLCKD), which has demonstrated positive outcomes in other neurological disorders that share similarities with FM, could be a plausible treatment strategy because of both weight loss and the effects of ketones on the nervous system. Their pilot study of 18 female participants demonstrated a beneficial impact of various FM measures with a personalized VLCKD. They noted that, despite weight loss, BMI did not necessarily track patient-reported outcomes over time, leading them to surmise that benefits may also come from the pleiotropic effects of a ketogenic diet, such as neuroprotection and lower neuroinflammation. The effect of weight loss is, however, important to pain outcomes. Ward et al. demonstrated improved pain and functional mobility outcomes in a secondary analysis of 110 participants on a three-month calorie-restricted diet who achieved a 7.0 kg weight loss.

Polyunsaturated fatty acids (PUFAs) are essential fatty acids that must be obtained through the diet. They are involved in immune system function, with omega-6 PUFAs being the precursor to eicosanoids, which are generally pro-inflammatory, and omega-3 PUFAs being involved in the resolution of inflammation (29, 30). Omega-6 intake per day has risen from 2.7g/day in the 1960s to up to 21g/day (31) largely due to UPF made with vegetable oils (29), resulting in the increase of the omega 6:3 ratio from around 4:1 to over 20:1 (30). Diets high in omega-6 have also been shown to contribute to chronic pain (32–34) and may be responsible for the upregulation of nociception (33). An O6:O3 ratio >5 is associated with higher pain outcomes and functional limitations, as well as higher inflammatory biomarkers of systemic inflammation (16). In contrast, diets that improve this ratio by increasing omega-3 or decreasing omega-6 intake may improve pain outcomes (33). Dai et al. analyzed PUFA concentrations and reported pain from a GWAS database to assess causal relationships. Their results also demonstrated that higher omega-3 concentrations were associated with lower abdominal, pelvic, and lower back pain. Additionally, a higher omega 6:3 ratio was positively associated with abdominal and pelvic pain and supports findings from other authors of increased orofacial and lower back pain and headache (33).

We the editors hope that, collectively, this Research Topic sustains interest in investigating the potential role diet and functional foods may play in the treatment and management of chronic pain and stimulates new areas of inquiry for the betterment of patient outcomes.
